# Spiking models of interaural level difference encoding - beyond the rate subtraction code

**DOI:** 10.1186/1471-2202-15-S1-P200

**Published:** 2014-07-21

**Authors:** Martin Spencer, Bertrand Fontaine, Romain Brette

**Affiliations:** 1 Institut d’études de la cognition, École Normale Supérior, Paris, 75005, France; 2 Sorbonne Universités, UPMC Univ. Paris 06, UMR_S 968, Institut de la Vision, Paris, F-75012, France; 3 INSERM, U968, Paris, F-75012, France; 4 CNRS, UMR_7210, Paris, F-75012, France; 5 Laboratory of Auditory Neurophysiology, University of Leuven, Leuven, Belgium

## 

Interaural Level Difference (ILD) provides an important cue for the location of a sound source in the azimuthal plane. Typically, ILD decoding in the brainstem is modeled as a subtraction of spike rates, with inhibitory inputs from one ear subtracted from the excitatory inputs from the other [1-3]. The inferior colliculus (IC) is known to receive input from this circuit, and to encode the spatial location of sounds.

Recent experimental evidence suggests that inhibitory input for ILD doesn’t provide subtraction, but instead provides a gain adjustment [4]. In addition, the exact mechanism of the creation of spatial receptive fields in the inferior colliculus remains unclear, and may also be a gain mechanism [5]. The excitatory input to the IC from neurons that decode ILD may contain spike timing cues for location. These spike-timing cues may be initiated in the ILD encoding cells even if the cues are absent from the inputs from the cochlear nucleus. In this study we used a spiking neuron model to recreate and model the full circuit of ILD sensitivity, and explore both the issue of ILD decoding, and the representation of sound source location in the IC.

The auditory periphery was modeled as a gammatone filterbank which provided inputs directly to a leaky integrate-and-fire model representing the cells of the cochlear nucleus. These cells are known to lock to the envelope of the sound stimulus, and this behavior was recreated by low-pass filtering of the gammatone filterbank inputs to the cells, and use of a dynamic spike threshold mechanism [6]. The ILD sensitive cells and IC cells were both modeled as simple leaky integrate-and-fire neurons. The model was able to recreate important experimental results regarding ILD encoding cells, particularly the variation of sensitivity with source intensity, and successfully created spatial receptive fields like those found in the IC. The results will be helpful in the future understanding of the binaural mechanisms of the auditory brainstem.
